# Shooting darts: co-evolution and counter-adaptation in hermaphroditic snails

**DOI:** 10.1186/1471-2148-5-25

**Published:** 2005-03-30

**Authors:** Joris M Koene, Hinrich Schulenburg

**Affiliations:** 1 Department of Animal Ecology, Institute of Ecological Sciences, Vrije Universiteit, De Boelelaan 1085, 1081 HV Amsterdam, The Netherlands; 2 Department of Developmental and Behavioural Neurobiology, Faculty of Earth and Life Sciences, Vrije Universiteit, De Boelelaan 1085, 1081 HV Amsterdam, The Netherlands; 3 Department of Evolutionary Biology, Institute for Animal Evolution and Ecology, Westphalian Wilhelms-University, Hüfferstrasse 1, 48149 Münster, Germany; 4 Department of Animal Evolutionary Ecology, Zoological Institute, University of Tübingen, Auf der Morgenstelle 28, 72076 Tübingen, Germany

## Abstract

**Background:**

Evolutionary conflicts of interest between the sexes often lead to co-evolutionary arms races consisting of repeated arisal of traits advantageous for one sex but harmful to the other sex, and counter-adaptations by the latter. In hermaphrodites, these antagonistic interactions are at least an equally important driving force. Here, we investigate the evolution of one of the most striking examples of sexual conflict in hermaphrodites, the so-called shooting of love-darts in land snails. Stabbing this calcareous dart through the partner's skin ultimately increases paternity. This trait is obviously beneficial for the shooter, but it manipulates sperm storage in the receiver. Hence, an arms race between the love-dart and the spermatophore receiving organs may be expected.

**Results:**

We performed a detailed phylogenetic analysis of 28S ribosomal RNA gene sequences from dart-possessing land snail species. Both the Shimodaira-Hasegawa test and Bayesian posterior probabilities rejected a monophyletic origin of most reproductive structures, including the love-dart, indicating that most traits arose repeatedly. Based on the inferred phylogenetic trees, we calculated phylogenetically independent contrasts for the different reproductive traits. Subsequent principal component and correlation analyses demonstrated that these contrasts covary, meaning that correlated evolution of these traits occurred.

**Conclusion:**

Our study represents the first comprehensive comparative analysis of reproductive organ characteristics in simultaneous hermaphrodites. Moreover, it strongly suggests that co-evolutionary arms races can result from sexual conflict in these organisms and play a key role in the evolution of hermaphroditic mating systems.

## Background

Evolutionary conflicts of interest between the sexes have been convincingly demonstrated in species with separate sexes [1]. These sexual conflicts often give rise to traits that are advantageous for one sex but harmful to the other. If these detrimental effects are counteracted, a co-evolutionary arms race may ensue in which harmful traits and corresponding counter-adaptations arise repeatedly [2]. Such antagonistic interactions can bring about major changes in the mating behaviour, genital morphology, gametes and seminal products, potentially leading to speciation [2]. Similar arms races seem to occur in hermaphrodites, contrary to Darwin's conviction [3] that sexual selection cannot act in hermaphroditic organisms. In fact, theoretical modeling indicates that these processes can become even more extreme in hermaphroditic species (N.K. Michiels and J.M. Koene, unpublished data), mainly because within one mating simultaneous hermaphrodites gain paternity (male fitness) which can outweigh the loss in female fitness. Here we investigate the evolution of a most peculiar reproductive behaviour that occurs in simultaneously hermaphroditic land snails (Stylommatophora), the "shooting" of a so-called love-dart into the mating partner.

Several explanations have been offered for the evolution of the enigmatic dart shooting behaviour. The dart is made of calcium carbonate and has therefore been proposed to serve as a nuptial gift of calcium for the production of eggs [4,5]. However, in *Cantareus aspersus *(previously *Helix aspersa*) the dart does not contain enough calcium to significantly contribute to egg production and darts are only rarely incorporated by the recipient [6]. Likewise, in other investigated species darts are even retained by shooters to be reused on the next mate [[7,8], J.M. Koene and S. Chiba, unpublished data). Therefore two other hypotheses have been put forward. In the female choice hypothesis the dart represents a sexual signal and recipients select on dart shooting effectiveness [5,9]. The important prediction of this hypothesis is that this can only be beneficial for the recipient if the dart is shot consistently by individuals (assuming that shooting ability is heritable). Tests in *C. aspersus *do not support this, because dart shooting of individually-identified animals in consecutive matings is unpredictable (G-test: *N *= 29 snails, *df *= 1, *G *= 6.745, *P *< 0.01; J.M. Koene, unpublished data). Besides, in *Arianta arbustorum *dart shooting seems to be an optional component of courtship [10].

**Figure 1:**
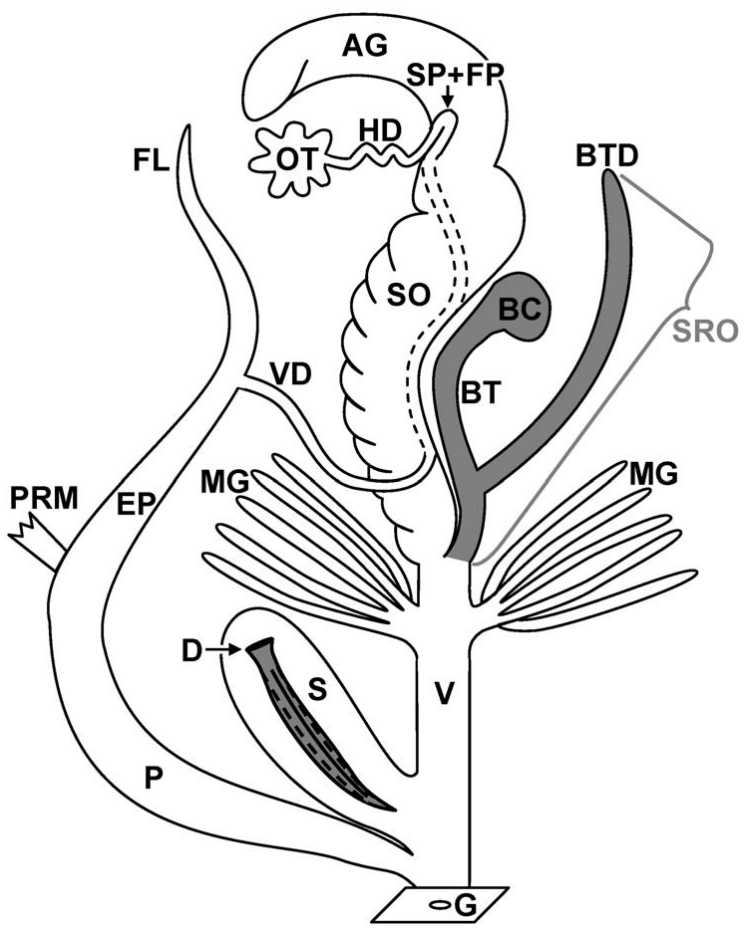
**Schematic morphological drawing of the reproductive morphology of a land snail with one dart and a diverticulum. **The love-dart (D) is produced and stored in the stylophore (S, often called dart sac) and shot by a forceful eversion of this organ. The mucus glands (MG) produce the mucus that is deposited on the dart before shooting. The penis (P) is intromitted to transfer the spermatophore. The sperm container is formed in the epiphallus (EP), while the spermatophore's tail is formed by the flagellum (FL). When a bursa tract diverticulum (BTD) is present, the spermatophore is received in this organ. Together with the bursa tract (BT) and bursa copulatrix (BC) these form the spermatophore-receiving organ (SRO, indicated in grey), which digest sperm and spermatophores. Sperm swim out via the tail of the spermatophore to enter the female tract and reach the sperm storage organ (SP, spermathecae) within the fertilization pouch (FP)-spermathecal complex. Other abbreviations: AG, albumen gland; G, genital pore; HD, hermaphroditic duct; OT, ovotestis; PRM, penis retractor muscle; SO, spermoviduct; V, vaginal duct; VD, vas deferens.

In the last hypothesis, the dart is used to manipulate the mating partner and can thus cause a sexual conflict [11]. This latter hypothesis seems most consistent with findings in the common garden snail *C. aspersus*, the species in which dart shooting and copulatory behaviour has been extensively studied. During courtship, the stylophore (dart sac) is everted and the single calcareous dart is pierced into the partner. Both mating partners normally shoot a dart before their penises are simultaneously intromitted. During intromission, spermatophores are exchanged and transferred into the partner's receiving organ, either directly into the bursa copulatrix or in an associated diverticulum (Figure 1). To avoid digestion in the spermatophore receiving organ (SRO), sperm have to actively swim out via the spermatophore's tail (formed by the flagellum of the penis) into the vaginal duct to reach the sperm storage site (the spermathecae) [12]. Previous work on *C. aspersus *has demonstrated that the shooting of the love-dart serves to introduce an allohormone [13-15], produced by associated glands, into the blood of the partner [16]. This allohormone inhibits digestion of sperm [17], which results in more of the donated sperm reaching the spermathecae [18] and fertilizing eggs [19,20]. This manipulation of the sperm storage process caused by darts can have a negative effect on the recipient's (reproductive) fitness because of interference with cryptic female choice.

Interestingly, love-darts display an astonishing diversity between species, both in number and shape, ranging from several simple cone-shapes to one elaborately bladed structures [21,22]. The most elaborate darts show surface enlargement with blades that is likely to enhance the transfer of gland product. We therefore predict that dart elaboration should covary with allohormone production, which should be mirrored in the gland morphology as surface enlargement. Moreover, the dart specializations that enhance gland product transfer are potentially more successful at manipulating fertilization. If so, adaptations to counteract this effect are expected, which could give rise to a co-evolutionary arms race. These predictions are tested here using a comparative analysis of dart-possessing land snail species. Note that we are only focussing on the Helicoidea superfamily; we do not include species with non-homologous dart-like structures (see also [22]). Because the phylogeny of land snails is heavily based on reproductive morphology [23,24], we first reconstructed an independent phylogeny based on part of the 28S ribosomal RNA (rRNA) gene [25]. Our findings represent the first comprehensive comparative analysis of reproductive organ characteristics in simultaneously hermaphroditic animals and are consistent with co-evolution and counter-adaptation predicted by sexual conflict theory.

## Results

### Reproductive organ morphologies

**Figure 2:**
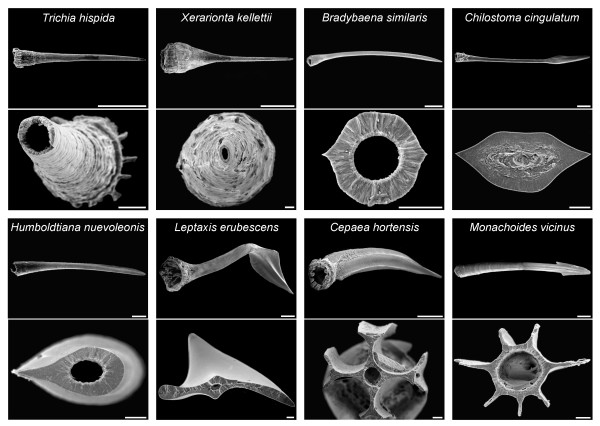
**Diversity of love-darts. **The different shapes of love-darts are illustrated with electron microscopic photographs of side views and cross sections of darts from different species. Scale bars indicate 500 μm for side views and 50 μm for cross-sections.

**Figure 3:**
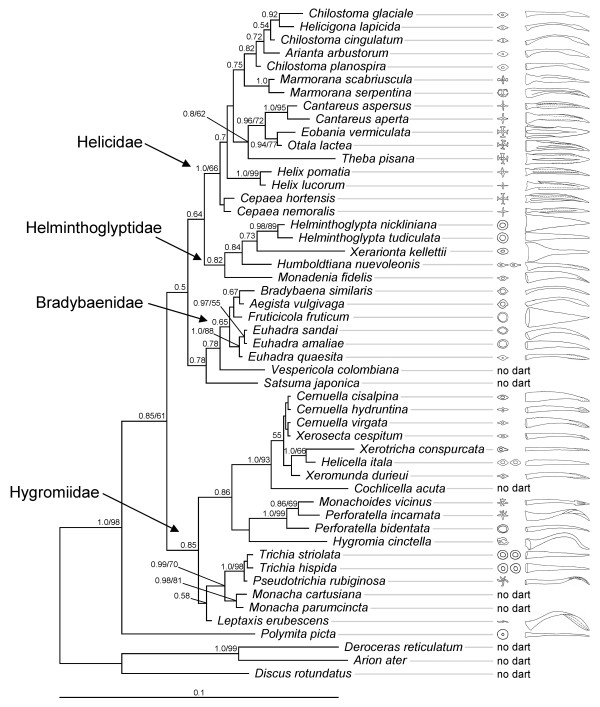
**Phylogeny of land snails and their love-darts. **Cross-section and side views of the darts are shown. For comparability, the line drawings are all at the same size. When two cross-sections are shown, that species possesses two functional darts. The shown phylogeny was obtained by Bayesian inference (BI). Branch lengths correspond to the number of substitutions per site (see scale bar). Maximum likelihood (ML) produced an almost identical tree (see Results). Clade support is given next to the nodes such that values before slashes refer to BI posterior probabilities above 0.5 and values behind slashes to ML bootstrapping results above 50.

Within the land snails that possess love-darts, there is a large diversity in reproductive structures and the darts themselves provide an impressive range of shapes. There are species with one dart, while others have several or none. Darts of some species have a simple cone-shape, whereas others show surface enlargement with blades. Additionally, darts vary from straight to curved and contorted. This variety in dart shapes is illustrated in the electron microscopic photographs in Figure 2 and the line drawings in Figure 3. We found similar levels of variation between species in the other reproductive organs. These differences include the number, relative size and placement of both functional and vestigial stylophores; the number, relative size, type of branching, and placement of the glands; the presence, relative length and placement of the diverticulum in the SRO; and the presence and relative length of the flagellum.

### Phylogenetic analysis

Both ML and BI yielded a single optimal phylogenetic tree (see Figure 3 for the BI tree). These two trees had almost identical topologies. The only differences consisted of the exact position of *Fruticicola fruticum *within the Bradybaenidae, of *Leptaxis erubescens *within the Hygromiidae, the relationship of the four major lineages of the Helicidae, and the position of *Cernuella cisalpina*, *C. hydruntina*, *C. virgata*, and *Xerosecta cespitum *in relation to each other. Moreover, although BI produced high support values for a much larger number of clades than ML bootstrapping, the clades with high ML bootstrap scores also always had high BI posterior probabilities (Figure 3). Furthermore, the four main families were all correctly identified (Figure 3). The only exception being that *Polymita picta *was not grouped with the other species of the Helminthoglyptidae. However, this taxon is found at the end of a comparatively long branch. Hence, its position at the base of the superfamily Helicoidea could be due to long branch attraction to the outgroup and may therefore be unreliable.

Both the SH test and BI posterior probabilities indicate a multiple origin of most reproductive organ characteristics (Table 1). Namely, a single origin was always rejected by BI posterior probabilities except for the presence of the diverticulum when monophyly was assumed within each of the four main families (Helicidae, Bradybaenidae, Helminthoglyptidae, Hygromiidae). The SH test did not confirm a single origin of the number of blades on the darts, the number of stylophores and glands, and the shape of glands if monophyly was assumed across all land snails. However, when monophyly was only hypothesized to occur within the four main families, then the SH test only rejected it for the stylophore number and the shape of the glands.

**Table 1:** Assessment of the monophyletic origin of reproductive organ characteristics.

H_0_	-ln L	Δ	*P*_rell_	*P*_BI_
**Monophyly assumed across all snails**				
Optimal tree	3240.98			
Dart number	3330.76	89.78	0.031	<0.001*
Blade number	3538.66	297.68	<0.001*	<0.001*
Presence of perpendicular blades	3283.10	42.12	0.399	<0.001*
Stylophore number	3450.29	209.31	<0.001*	<0.001*
Gland number	3492.69	251.71	<0.001*	<0.001*
Shape of glands	3705.34	464.36	<0.001*	<0.001*
Presence of flagellum	3326.69	85.71	0.044	<0.001*
Presence of diverticulum	3245.56	4.58	0.905	0.002*
**Monophyly assumed within families**				
Optimal tree	3240.98			
Dart number	3295.14	54.16	0.234	<0.001*
Blade number	3351.62	110.64	0.012	<0.001*
Presence of perpendicular blades	3283.11	42.13	0.399	<0.001*
Stylophore number	3386.55	145.57	<0.001*	<0.001*
Gland number	3373.91	132.92	0.005	<0.001*
Shape of glands	3455.91	214.93	<0.001*	<0.001*
Presence of flagellum	3269.58	28.60	0.574	<0.001*
Presence of diverticulum	3240.98	0	0.962	0.843

The following ancestral states were assumed: Zero darts, no blades on the dart, no perpendicular blades on the blades, two functional and two vestigial stylophores, many tubular glands, a simple gland shape, and absence of the flagellum of the penis and diverticulum of the spermatophore-receiving organs. Abbreviations: -ln L, negative natural logarithmic likelihood value for the hypothesis, as calculated from a constrained tree using ML in PAUP*. **Δ**, difference from the optimal tree; *P_rell_*, probability according to the Shimodaira-Hasegawa test; *P_BI_*, Bayesian posterior probability; *, Significance after Bonferroni correction.

### Principal component and correlation analysis

The overall PCA (Table 2) that was performed on the BI dataset revealed that most PICs loaded positively and significantly on the first PC axis (at *P *< 0.15 or *P *< 0.05 (see [26])) and explained 32.5% of the total variance. This result suggests that the traits all covary. To examine this correlated evolutionary pattern in more detail we performed separate PCAs on the data for darts, stylophores, glands, and spermatophore receiving organs (Additional file 1) followed by a correlation analysis. These PCAs permitted for successful reduction of the variable number per trait to two PCs. For each organ the first two PCs explained over 70% of the variation for each trait, regardless of the tree used for PIC calculation (Additional file 1). The interpretation of the PCs can be deduced from the inferred eigenvectors (Additional file 1) and is mentioned between brackets in the following description of the results from the correlation analyses.

**Table 2:** Principal component analysis on phylogenetically independent contrasts of love-dart and reproductive morphology data.

	Component loadings			
	
Data	PC1	PC2	PC3	PC4
**Love-dart**				
Number of darts	0.807*	-0.212	-0.262	0.050
Number of blades	0.492†	0.544	0.303	0.318
Length of blades	0.664*	0.381	0.517	0.158
Curvature	0.534*	0.169	0.127	0.668
**Functional stylophore(s)**				
Number	0.781*	-0.204	-0.379	-0.219
Relative size	0.411†	0.498†	-0.447	0.393
Placement on vaginal duct	0.536*	0.053	-0.578	-0.286
**Vestigial stylophore(s)**				
Number	0.527†	-0.775	0.125	0.013
Relative size	0.361†	-0.655	0.258	0.180
Placement on vaginal duct	0.527†	-0.753	0.165	0.106
**Glands**				
Number	0.709*	-0.339	-0.050	-0.225
Relative size	0.472	0.592	-0.275	-0.089
Type of branching	0.275	0.360	-0.277	0.189
Placement	0.871*	-0.133	-0.050	0.258
**Diverticulum of spermatophore receiving organ**				
Length relative to bursa tract	0.457	0.359	0.216	-0.470
Placement	0.554*	0.308	0.431	-0.342
Relative size	0.184*	0.454	0.303	-0.167
**Flagellum**				
Relative length	0.614*	0.141	0.138	-0.429

The independent contrasts are based on the trees reconstructed with BI without consideration of five cases of phylogenetic uncertainty. The table shows the loadings for each variable in the different principal components (PC1 to PC4). Collectively the principal components explain over 70% of the total variance in the data set (respectively, 32.5%, 19.3%, 9.6%, and 9.0 %). Tests of significance of variable loadings represent frequency of loadings different in sign to the ones observed, among 1000 bootstrap replicate analyses corrected for axis reversals (*, P < 0.05; †, P < 0.15).

**Figure 4:**
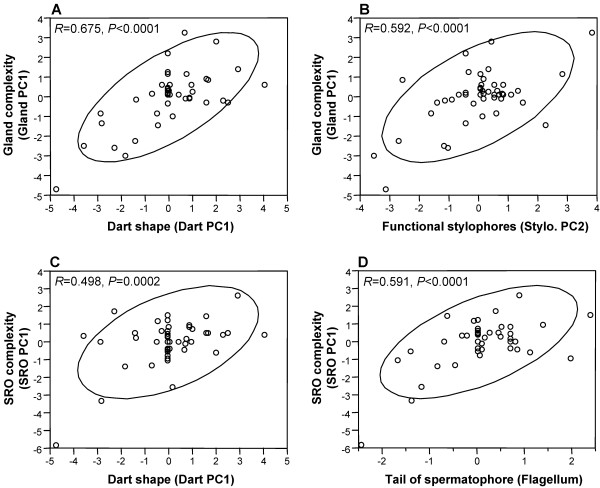
**Graphs illustrating co-evolution and counter-adaptation. ***A*, Correlated evolution of the darts and glands. *B*, Correlated evolution between stylophores and glands. *C*, Counter-adaptation of diverticulum in response to changes in the dart. *D*, Counter-adaptation of the spermatophore's tail (formed by the flagellum) in response to changes in SRO complexity. The principal components used to create the graphs are those obtained from the independent contrasts based on the BI tree without phylogenetic uncertainty. The ellipses represent the 95% confidence interval, Pearson correlations (*R*) and *P*-values are indicated in each graph.

The correlation analyses in Table 3 shows the relationships between the PCs for the different traits based on the BI tree without phylogenetic uncertainty. Significant correlations indicate co-evolution between organs. The correlations in Figure 4 are also based on the BI tree without phylogenetic uncertainty, but essentially identical results were obtained for the PICs calculated from the other phylogenetic trees as well as the raw data (Additional file 2). Additionally, we only show results for the PCAs based on all the variables; the results were essentially identical when only the significantly loading variables were included (data not shown). Most importantly, there are significantly positive correlations between Dart PC1 (dart shape) and Gland PC1 (gland complexity) (Figure 4A); Dart PC2 (dart number) and Stylo. PC1 (vestigial stylophores); Gland PC1 (gland complexity) and Stylo. PC1 as well as Stylo. PC2 (functional stylophores) (Figure 4B). Additionally, there is a significantly negative correlation between Gland PC2 (gland shape) and Stylo PC1 (vestigial stylophores). Furthermore, the SRO PC1 (SRO complexity) shows significant positive correlations with Dart PC1 (dart shape) and Gland PC1 (gland complexity) (Figure 4C). Finally, we also found that the length of the flagellum of the penis (which forms the spermatophore's tail) positively correlates with SRO PC1 (Figure 4D) as well as with Gland PC1 (gland complexity).

**Table 3:** Pearson correlations (below diagonal) and their significance (above diagonal) for the comparison of principal components (PC) and flagellum.

	**BI**								
	**Dart PC1**	**Dart PC2**	**Stylo. PC1**	**Stylo. PC2**	**Gland PC1**	**Gland PC2**	**SRO PC1**	**SRO PC2**	**Flag. length**
**Dart PC1**		1.0000	0.0711	0.0120	<0.0001*	0.7586	0.0002*	0.9030	0.0136
**Dart PC2**	0.000		<0.0001*	0.0330	0.0063	0.1482	0.2417	0.3583	0.3122
**Stylo. PC1**	0.257	0.631		1.0000	<0.0001*	0.0006*	0.6756	0.0942	0.0459
**Stylo. PC2**	0.353	0.302	0.000		<0.0001*	0.0609	0.0663	0.9023	0.0311
**Gland PC1**	0.675	0.381	0.532	0.592		1.0000	0.0024*	0.1921	0.0002*
**Gland PC2**	0.045	-0.208	-0.468	0.267	0.000		0.2786	0.8307	0.8816
**SRO PC1**	0.498	-0.169	0.061	0.262	0.420	0.156		1.0000	<0.0001*
**SRO PC2**	0.018	-0.133	-0.239	0.018	-0.188	-0.031	0.000		0.2290
**Flag. length**	0.347	0.146	0.284	0.305	0.502	-0.022	0.591	-0.173	

Results are shown for the analysis based on PICs calculated from the BI without phylogenetic uncertainty. *, Significance after Bonferroni correction.

One potential problem of this detailed analysis is that our character coding may produce a bias in the data when organs are absent, because then the organ itself as well as all related traits are scored as zero. However, this does not have a strong effect on the analysis of darts, stylophores, glands and flagellum, where there are only very few taxa in which the respective organ is absent. The diverticulum is not always present in the SRO, but in a correlation analysis of the data only including presence/absence of this organ (thus removing all irrelevant zero's) the same combinations were significant.

## Discussion

Sexual conflict can cause counter-adaptive co-evolution between male and female reproductive organs. Although such conflicts may become costly, when looking at only one species these costs usually remain hidden because the mating partners are well adapted to each other [27,28]. Therefore, an inter-species comparison is required to reliably reveal patterns of counter-adaptive co-evolution driven by sexual conflict [29]. The results from our detailed comparative analysis are indeed most consistent with the presence of a co-evolutionary arms race in simultaneous hermaphrodites, which has generated a diversity of dart shapes and reproductive organ morphologies. This finding corroborates the theoretical prediction that sexual conflict and counter-adaptation can play a major role in the evolution of hermaphroditic mating systems and reproductive morphologies [30,31].

In detail, our findings provide evidence for both repeated and correlated evolution between traits associated with dart shooting and spermatophore receipt. The occurrence of both is considered convincing evidence for a co-evolutionary arms race. The reconstructed phylogenies that we used to test this, were estimated from the 28S rRNA gene with different tree reconstruction methods (see Methods). In agreement with previous studies [32-34], ML bootstrap values for the inferred clades are generally smaller than the BI posterior probabilities. Hence, BI support for the inferred clades may be overestimated. Although BI is considered to provide a highly consistent framework for phylogenetic inference, its reliability under different evolutionary scenarios and the interpretation of clade posterior probabilities is still under debate [32-34]. Nevertheless, in our study ML and BI produced highly similar trees. In addition, the results are consistent with morphology-based trees, for example all families form monophyletic groups except for the Helminthoglyptidae [23,24]. Hence, the consistency of results obtained with different methods and between molecular and morphology-based phylogenies suggests that the inferred phylogeny is generally robust.

Even though small discrepancies existed between the inferred phylogenies, they did not seem to have a large effect on the subsequent analyses (PCA and correlation analysis). These analyses produced consistent results regardless of the underlying topology or the consideration of phylogenetic uncertainty (Table 3 and Additional file 2). Furthermore, a comprehensive analysis of computer simulated data highlighted that in comparative analyses the consideration of phylogenies with a limited number of uncertainties still yields considerably more reliable results than no consideration of phylogenetic relationships [35].

Both the SH test and BI posterior probabilities reject a single origin of most reproductive structures when monophyly is assumed across all land snails, as was already suggested for the dart by Tompa [36]. When monophyly is assumed within each of the well-supported families (Helicidae, Bradybaenidae, Helminthoglyptidae, Hygromiidae) monophyly is still rejected in some cases but with less significance (Table 1). Because BI tends to overestimate reliability of inferred clades (i.e. very high posterior probabilities), it automatically underestimates support for clades, which are not represented in the inferred tree. In this case, hypotheses of their monophyly would be often incorrectly rejected. This could be the reason for our finding that BI posterior probabilities for the specific hypotheses of monophyly were almost always highly significant, even when the SH test statistics were clearly insignificant. Nonetheless, taken together the results strongly indicate that we are dealing with repeated evolution of most characteristics of the reproductive system.

Using phylogenetically independent contrasts (PICs), to account for phylogenetic affiliation of the taxa [37], we found clear evidence for correlated evolution. Namely, the overall principal component analysis demonstrates that correlated evolution across the PICs occurs on one co-evolutionary axis, meaning that the traits covary [26]. Examination of this pattern in more detail revealed that when darts become more elaborate the number of stylophores (and thus the number of darts, as previously suggested [21,22]) decreases and the complexity of the glands increases (Figure 4A,B). These co-evolutionary patterns lead us to conclude that the changes enhance the transfer of gland product and possibly improve sperm storage, provided that the gland products of the investigated species have similar effects as those seen in *C. aspersus *[17,19,18,20].

If inhibition of sperm digestion occurs, it will ultimately influence sperm storage and thus fertilization success [17,19,18,20], which is beneficial for the shooter and hence favoured by sexual selection. However, the receiver's fertilization processes are manipulated by the mucus while the dart itself causes damage and may increase infection risk. Consequently, counter-measures can be expected. Behavioural counter-adaptations to prevent dart receipt seem unlikely because the tactile information from contact with the partner's skin, which cannot be avoided during mating, is essential for dart shooting to occur [38]. Conversely, physiological or morphological counter-adaptations are possible, e.g. changes in allohormone receptor sensitivity, skin thickness, or female reproductive morphology. Here, we report morphological counter-adaptations in the spermatophore receiving organs (Figure 4C). The adaptations primarily entail the appearance and subsequent lengthening of a diverticulum. These changes increase the distance sperm need to travel to the spermathecae, presumably this hampering of sperm storage occurs to offset the increased sperm survival caused by the more elaborate darts and glands.

The latter change poses an additional complication because sperm are most successful at reaching the storage site when the spermatophore's tail is protruding into the vaginal duct [12]. The tail of the spermatophore is formed by the flagellum of the penis, which we used as a measure of the tail's length. The significant correlations of the flagellum (spermatophore's tail) with both the SRO and gland complexity can therefore also be interpreted as an indication of counter-adaptation.

The presented study provides a first step into understanding the diversity of love-darts in land snails and clearly raises several questions for future studies. For example, the previously available experimental data strongly suggest that dart shooting serves to manipulate the partner (manipulation- or sexual conflict hypothesis), whereas alternative hypotheses are not supported (nuptial gift- and female choice hypothesis; see background section). Although the manipulation hypothesis is entirely consistent with the results of the current study, it implies that dart shooting benefits the shooter, as previously demonstrated [13-20], and that it also negatively affects the receiver, for which no direct evidence is available yet. Hence, an important challenge for the future is to evaluate the possible costs of receiving a dart. In this context, our results also allow to choose closely related species with pronounced differences in dart morphology (e.g. *C. hortensis *and *C. nemoralis*) for a more detailed experimental study addressing the causes and consequences of reproductive organ diversity. The examination of variation among closely related species pairs and/or within species may additionally provide novel insights into the dynamics of the co-evolutionary adaptations. Finally, evidence is accumulating that not all species use their dart in the same way. Hence, additional behavioural data are pivotal for a full understanding of the evolution of these darts.

## Conclusion

We found support for both repeated and correlated evolution, which we consider compelling evidence for a co-evolutionary arms race. Furthermore, because empirical findings are most consistent with the manipulation hypothesis, we conclude that the observed co-evolutionary patterns result from a sexual conflict. This comparative study is the first of its kind in simultaneous hermaphrodites. The results strongly suggest that sexually antagonistic co-evolutionary interactions can play an equally important role in hermaphrodites as they do in organisms with separate sexes [29,39]. As such, sexually antagonistic co-evolution may provide an important driving force for the evolution of hermaphroditic mating systems and possibly even speciation. Moreover, it may also account for some of the other bizarre reproductive structures and behaviours found in hermaphrodites like gigantic penises [40] and penis biting [41] in land slugs, hypodermic insemination in tropical flatworms [42], and body piercing in earthworms [43].

## Methods

### Snails material

We obtained data from 51 land snail species from the four main dart-possessing families: the Helicidae, the Hygromiidae, the Helminthoglyptidae, and the Bradybaenidae. We also included one member of both the Polygyridae and Camaenidae, plus three outgroup taxa. Mature specimens of the species were collected by JMK or provided by colleagues (Additional file 3). Prior to fixation in 80% EtOH the animals were relaxed by drowning, which was also the standard protocol for specimens obtained from the malacological collection of the Academy of Natural Sciences of Philadelphia. In three cases, where we could not obtain snail material ourselves, we used previously published information on reproductive organ morphology [44] and DNA sequence data [25] (Additional file 3). In 21 other cases, because previously published DNA sequence data were already available [25], we only obtained data on reproductive organ characteristics (Additional file 1).

### Reproductive organ morphologies

We examined five different reproductive structures: darts, stylophores, glands, spermatophore receiving organs, and the flagellum of the penis (Figure 1). For this, adult specimens of each species were dissected to remove the reproductive tract. Subsequently, the reproductive organs were drawn using a *camera lucida*. These drawings were used to determine relative organ sizes, thus correcting for body size (see Additional file 4). The albumen gland, which is seasonally variable because it provisions the eggs, was not included when determining relative sizes. Only adult animals from one location with fully formed darts were included. To avoid damage of the darts, the stylophores were carefully cut out of the reproductive tracts and placed overnight in 1N NaOH, which dissolved all the tissue and mucus but left the dart intact. For cross-sections, darts were carefully broken in two. The intact and broken darts were consecutively prepared for electron microscopy by placing them on small aluminium plates with an electrically conducting adhesive (Leit-Tab, Plano). They were then coated with gold using a Metalloplan (Leitz). The darts were placed under a scanning electron microscope (S-530 SEM, Hitachi) and photos were taken. The characteristics of the reproductive structures were scored as ranks in order of complexity, based on the traditional taxonomic literature (e.g. [24]) (Additional file 4).

### Molecular data

The phylogeny of the snails was examined using an analysis of the 5' end of the 28S rRNA gene. DNA was isolated from snails using a CTAB-based protocol [45]. In detail, snail tissue was ground up with a pestle in 400 μl CTAB buffer (2% (w/v) Cetyl-trimethyl-ammonium-bromid, 0.1 M Tris-HCL pH 8.0, 0.02 M EDTA, 1.4 M NaCl, 0.2% (v/v) β-Mercaptoethanol). Tissue was further digested by addition of 4 μl Proteinase K (10 mg/ml) and incubation at 50°C overnight. DNA was extracted by addition of 2 volumes chloroform:isoamylalcohol (24:1) and centrifugation at 13.000 rotations per minute (rpm) for 15 min. The DNA containing supernatant was recovered and DNA was precipitated by addition of 2/3 volumes of 100% isopropanol, incubation of the mixture at -20°C for 1 h, and subsequent centrifugation at 13.000 rpm for 30 min. The DNA pellet was finally washed in 70% ethanol, left to dry and resuspended in 50–100 μl sterile Millipore H_2_O. The 5' end of the 28S rRNA gene was amplified via PCR, using primers designed in conserved regions of the ribosomal cistron of molluscs (Additional file 5). Amplification was performed under standard reaction conditions: 1 U Taq Polymerase (Promega Ltd.), 50 mM KCl, 10 mM Tris-HCl pH 9.0, 0.1% Triton X-100, 2.5 mM MgCl_2_, 0.2 mM of each dNTP, 0.5 μM of each Primer. The following cycling profile was used: 5 min at 95°C, followed by 35 cycles of 20 sec at 95°C, 30 sec at 62.5°C and 1 min at 70°C, and a final extension period of 10 min at 70°C. PCR products were purified using Microcon-50 microconcentrators (Millipore Ltd). DNA sequencing was subsequently performed with the reverse PCR primer and additional internal primers (Additional file 5), using the ABI Prism BigDye Terminator Cycle Sequencing Kit (Applied Biosystems Ltd) and visualization of results on an ABI310 Genetic Analyser (Applied Biosystems Ltd). All sequences are deposited at the EMBL database under accession numbers AJ550953 to AJ550982.

### Phylogenetic tree reconstruction

The DNA sequence alignment (EMBL: ALIGN_000524) was produced with CLUSTALW, using default settings [46], and was subsequently adjusted by eye in cases of obvious errors (e.g. large end gaps) using the program BIOEDIT, version 5.0.9 [47]. The alignment contained 51 taxa and 740 positions, of which 169 were variable (22.84%). All phylogenetic analyses were based on either conventional maximum likelihood (ML) as implemented in the program PAUP*, version 4, beta 10 [48] or Bayesian inference (BI) as implemented in the program MRBAYES, version 2.01 [49]. Both methods permit specification of substitution models to correct for multiple hits and provide a consistent statistical framework for hypothesis testing [50,51]. The Tamura-Nei substitution model with gamma-distributed rate heterogeneity across sites and a proportion of invariable sites (TN-Γ-I) was found to be optimal for the data with the help of likelihood ratio tests following the procedure outlined by Huelsenbeck and Crandall [52] and using the program MODELTEST, version 3.06 [53]. The TN-Γ-I model was employed for all subsequent ML analysis. Since the program MRBAYES currently does not support TN-Γ-I, BI was based on the next more complex model available, the general time reversible substitution model with rate heterogeneity across sites (GTR-Γ).

For ML tree estimation, parameters of the substitution model were first optimized using a maximum parsimony (MP) tree, inferred with a heuristic search via branch-swapping by tree bisection and reconnection (TBR). These parameter estimates were then employed in the ML tree search, using the heuristic search options and the MP tree as a starting topology for branch-swapping by TBR [for a similar approach, see [45]]. Nodal support was inferred via non-parametric bootstrapping [45] using the same ML settings and 200 replicate data sets. Specific hypotheses on the origin of reproductive organ characteristics were assessed with the Shimodaira-Hasegawa (SH) test [55]. For these tests, trees were first calculated with specified topological constraints, which each represented one of the hypotheses of interest. Tree calculation was again based on ML and the above settings. Thereafter, trees with topological constraints were compared with the optimal topology using the SH test statistics, inferred with the RELL bootstrap option, as implemented in PAUP* (see [56]).

BI was based on the Markov Chain Monte Carlo approach, using 500,000 generations, four chains (one cold and three heated), each started with a random tree, and saving of trees every 10 generations. Stationarity was reached after 40,000 generations. Calculation of consensus trees and summary statistics was thus based on trees obtained from only subsequent generations. BI was performed twice, using the same settings, and produced qualitatively identical results (identical tree topology, highly similar branch lengths and nodal support values) (see [49]). The monophyletic origin of reproductive organ characteristics was evaluated with the help of the inferred posterior probability of the respective clades [57].

### Independent contrast and principal component analysis

The taxa included are related to each other at different degrees. Hence, the data obtained for reproductive organ characteristics are not independent, resulting in increased type I errors in comparisons between taxa [37,58,59]. To correct for phylogenetic affinity, we calculated phylogenetically independent contrasts (PICs) using CONTRAST 3.6 [37]. We treated variables as continuous since the scores for each of them were ranked in order of complexity (for similar approaches see [60,61]). PICs were inferred from four tree topologies: the BI and the ML tree, both either with or without consideration of five cases of phylogenetic uncertainty. These cases refer to differences between BI and ML trees, which also lack support from BI posterior probabilities and ML bootstrapping. Because these differences are characterized by short branches, phylogenetic uncertainty was taken into account by setting branches to zero. This essentially produces hard polytomies, which is equivalent to a very rapid radiation rate (see [59] for a similar approach). Adequate standardization of contrasts was confirmed by plotting the absolute values of contrasts against their standard deviations [62]. Note that we decided against the approach of Pagel [63], as implemented in the program CAIC [64], to take account of phylogenetic uncertainty. This method relies on the *a-priori *specification of a predictor variable, which is not applicable for our data set.

To assess the main pattern of evolutionary covariance of the traits, we first performed a single principle component analysis (PCA) on all the PICs from the BI tree without phylogenetic uncertainty. Significance of variable loadings was tested by determining the frequency of loadings different in sign to the ones observed, among 1000 bootstrap replicate analyses (generated with SYSTAT 10.2) corrected for axis reversals [26]. We then also examined correlated evolution among pairwise compared traits, in order to obtain more fine-scale information about the co-evolutionary pattern. For this, we first used PCA to reduce the number of related PIC variables that jointly characterize a specific trait. Thus, separate principal components (PC) were created for the dart, stylophores, glands, and SRO. For each of these organs the PCs, which explained over 70% of the variance, were compared in a correlation analysis. PCA and correlation analysis was applied to the raw data and to PICs resulting from the four trees (see above), using the program JMP 4.0.2 (SAS Institute Inc.).

## Abbreviations

BI, Bayesian inference; ML, Maximum likelihood; MP, Maximum parsimony; PC, Principal component; PCA, Principal component analysis; PIC, phylogenetically independent contrasts SH, Shimodaira-Hasegawa test; SRO, spermatophore receiving organ; TBR, Tree bisection and reconnection.

## Authors' contributions

All the snail material and reproductive organ morphologies were collected and/or obtained by JMK. The molecular data collection was performed by JMK, while HS did the phylogenetic tree reconstruction and monophyly testing. HS also provided the independent contrasts, that were used by JMK in the principal component and correlation analyses. JMK and HS wrote the paper.

## Supplementary Material

Additional File 1 **Principal component analyses on raw data and phylogenetically independent contrasts of love-dart and reproductive morphology data. **The independent contrasts are based on the raw data and the three alternative trees (see Methods), namely BI or ML with and without consideration of five cases of phylogenetic uncertainty. The ML tree topologies with and without phylogenetic uncertainty were identical. The table shows the eigenvectors for each variable in the different principal components (PC). These vectors are a measure for the weight of the variable in the PC. The eigenvalue and % variance are given for each PC. These values express how much of the total variance in the data is explained by that PC. Click here for file

Additional File 2 **Matrix of Pearson correlations (below diagonal) and their significance (above diagonal) for the comparison of the different principal components (PC) and the flagellum based on the raw data and the three other phylogenetic trees. **Results are shown for the raw data and the analyses based on PICs calculated from the BI with phylogenetic uncertainty and the ML trees either with or without phylogenetic uncertainty. Note that the ML trees with or without uncertainty are identical. *, Significance after Bonferroni correction. Click here for file

Additional File 3 **Information about the location and collectors of the species used in the analysis. **Numbers behind collector names represent the catalog number in the malacological collection of the Academy of Natural Sciences of Philadelphia. The EMBL accession numbers for the new and published DNA sequences are also included. Published sequences all derive from Wade et al. 2001 [25]. ‡, The specimens from this location were used for sequencing; § for these species the DNA sequence were available from EMBL but no specimens could be obtained for investigation. Therefore, the morphology data were based on Azuma 1995 [44]. Click here for file

Additional File 4 **Description of the data scored for the different reproductive organ characteristics. **When traits are absent these are scored as zero. All relative sizes were measured on the camera lucida drawings. When the size is indicated relative to the reproductive organs this refers to the posterior reproductive organs (excluding the albumen gland) thus making sure that they are independent of the size of the penis and bursa tract. Click here for file

Additional File 5 **The sequences for PCR and sequencing primers. **Primers JOR58F, JOR59F, and JOR28R850 were used for PCR, whereby JOR59F was employed as the forward primer for *Cernuella cisalpina *and *Euhadra quaesita*, and JOR58F for all remaining species. Primers JOR28F50, JOR28F400, JOR28R401, JOR28F600, JOR28R601, and JOR28R850 were employed in sequencing reactions. Click here for file
