# Supplementary material for: Shooting darts: co-evolution and counter-adaptation in hermaphroditic snails
Source: BMC Evol Biol. 2005 Mar 30;5:25. doi: 10.1186/1471-2148-5-25 (PMC1080126; doi:10.1186/1471-2148-5-25)
Supplement: Additional File 1 — Principal component analyses on raw data and phylogenetically independent contrasts of love-dart and reproductive morphology data. The independent contrasts are based on the raw data and the three alternative trees (see Methods), namely BI or ML with and without consideration of five cases of phylogenetic uncertainty. The ML tree topologies with and without phylogenetic uncertainty were identical. The table shows the eigenvectors for each variable in the different principal components (PC). These vectors are a measure for the weight of the variable in the PC. The eigenvalue and % variance are given for each PC. These values express how much of the total variance in the data is explained by that PC. [file 1471-2148-5-25-S1.pdf]

Additional file 1. Principal component analyses on raw data and phylogenetically independent contrasts of love-dart and reproductive morphology data.

	Raw		BI		BI with phylogenetic uncertainty		ML with or without phylogenetic uncertainty	
**Love-dart variables**	**Dart PC1**	**Dart PC2**	**Dart PC1**	**Dart PC2**	**Dart PC1**	**Dart PC2**	**Dart PC1**	**Dart PC2**
Number	0.298	0.882	0.359	0.884	0.351	0.876	0.333	0.912
Number of blades	0.586	-0.270	0.544	-0.345	0.550	-0.342	0.536	-0.357
Length of blade	0.545	-0.350	0.549	-0.306	0.551	-0.321	0.562	-0.203
Curve	0.521	0.165	0.523	0.074	0.520	0.110	0.535	0.003
**Eigenvalue**	**2.320**	**0.933**	**2.416**	**0.828**	**2.379**	**0.851**	**2.404**	**0.853**
**%Variance**	**58.0**	**23.3**	**60.4**	**20.7**	**59.5**	**21.3**	**60.1**	**21.3**
**Gland variables**	**Gland PC1**	**Gland PC2**	**Gland PC1**	**Gland PC2**	**Gland PC1**	**Gland PC2**	**Gland PC1**	**Gland PC2**
Number	0.626	-0.350	0.538	-0.534	0.548	-0.523	0.510	-0.574
Size	0.373	0.537	0.447	0.429	0.437	0.426	0.453	0.430
Shape	0.119	0.760	0.277	0.712	0.257	0.727	0.299	0.679
Placement	0.675	-0.106	0.659	-0.155	0.665	-0.130	0.667	-0.158
**Eigenvalue**	**1.963**	**1.248**	**1.818**	**1.198**	**1.798**	**1.172**	**1.740**	**1.239**
**%Variance**	**49.1**	**31.2**	**45.4**	**29.9**	**45.0**	**29.3**	**43.5**	**31.0**
**Stylophore variables**	**Stylo. PC1**	**Stylo. PC2**	**Stylo. PC1**	**Stylo. PC2**	**Stylo. PC1**	**Stylo. PC2**	**Stylo. PC1**	**Stylo. PC2**
**Functional stylophore(s):**								
Number	0.425	0.356	0.389	0.461	0.384	0.468	0.349	0.487
Size	0.005	0.668	-0.058	0.575	-0.065	0.568	-0.135	0.578
Placement	0.290	0.542	0.180	0.590	0.166	0.594	0.048	0.622
**Vestigial stylophore(s):**								
Number	0.525	-0.193	0.562	-0.102	0.565	-0.091	0.562	-0.006
Size	0.449	-0.219	0.437	-0.287	0.440	-0.289	0.479	-0.201
Placement	0.507	-0.221	0.553	-0.126	0.555	-0.115	0.559	-0.040
**Eigenvalue**	**3.166**	**1.746**	**2.898**	**1.746**	**2.888**	**1.739**	**2.967**	**1.809**
**%Variance**	**52.8**	**29.1**	**48.3**	**29.1**	**48.1**	**29.0**	**49.4**	**30.1**

Diverticulum variables	SRO PC1	SRO PC2	SRO PC1	SRO PC2	SRO PC1	SRO PC2	SRO PC1	SRO PC2
Length (relative to bursa tract)	0.602	-0.311	0.635	-0.288	0.636	-0.297	0.667	-0.238
Placement	0.591	-0.445	0.627	-0.350	0.631	-0.331	0.668	-0.230
Length (relative to organs)	0.536	0.840	0.451	0.892	0.444	0.896	0.331	0.944
**Eigenvalue**	**2.174**	**0.528**	**1.681**	**0.827**	**1.664**	**0.837**	**1.490**	**0.940**
**%Variance**	**72.5**	**17.6**	**56.0**	**27.6**	**55.5**	**27.9**	**49.7**	**31.3**

The independent contrasts are based on the raw data and the three alternative trees (see Methods), namely BI or ML with and without consideration of five cases of phylogenetic uncertainty. The ML tree topologies with and without phylogenetic uncertainty were identical. The table shows the eigenvectors for each variable in the different principal components (PC). These vectors are a measure for the weight of the variable in the PC. The eigenvalue and % variance are given for each PC. These values express how much of the total variance in the data is explained by that PC.
