# Supplementary material for: Shooting darts: co-evolution and counter-adaptation in hermaphroditic snails
Source: BMC Evol Biol. 2005 Mar 30;5:25. doi: 10.1186/1471-2148-5-25 (PMC1080126; doi:10.1186/1471-2148-5-25)
Supplement: Additional File 2 — Matrix of Pearson correlations (below diagonal) and their significance (above diagonal) for the comparison of the different principal components (PC) and the flagellum based on the raw data and the three other phylogenetic trees. Results are shown for the raw data and the analyses based on PICs calculated from the BI with phylogenetic uncertainty and the ML trees either with or without phylogenetic uncertainty. Note that the ML trees with or without uncertainty are identical. *, Significance after Bonferroni correction. [file 1471-2148-5-25-S2.pdf]

Additional file 2. Matrix of Pearson correlations (below diagonal) and their significance (above diagonal) for the comparison of the different principal components (PC) and the flagellum.

Raw data									
	**Dart PC1**	**Dart PC2**	**Stylo. PC1**	**Stylo. PC2**	**Gland PC1**	**Gland PC2**	**SRO PC1**	**SRO PC2**	**Flag. length**
**Dart PC1**		1.0000	0.2587	0.0002*	<0.0001*	0.0430	0.0011*	0.4666	0.0022*
**Dart PC2**	0.000		<0.0001*	0.1861	0.0009*	0.1333	0.1459	0.8554	0.6095
**Stylo. PC1**	0.161	0.688		1.0000	<0.0001*	0.0061	0.1833	0.2579	0.2492
**Stylo. PC2**	0.501	0.188	0.000		<0.0001*	0.0046*	0.1214	0.2866	0.0247
**Gland PC1**	0.618	0.451	0.669	0.570		1.0000	0.4722	0.8503	0.0048*
**Gland PC2**	0.285	-0.213	-0.379	0.391	0.000		0.0002*	0.8842	0.0152
**SRO PC1**	0.443	-0.207	-0.189	0.220	0.103	0.499		1.0000	<0.0001*
**SRO PC2**	0.104	-0.026	-0.161	0.152	-0.027	-0.021	0.000		0.4807
**Flag. length**	0.419	0.073	0.164	0.314	0.389	0.338	0.662	-0.101	

BI with phylogenetic uncertainty									
	**Dart PC1**	**Dart PC2**	**Stylo. PC1**	**Stylo. PC2**	**Gland PC1**	**Gland PC2**	**SRO PC1**	**SRO PC2**	**Flag. length**
**Dart PC1**		1.0000	0.0850	0.0205	<0.0001*	0.6273	0.0002*	0.8955	0.0173
**Dart PC2**	0.000		<0.0001*	0.0162	0.0039*	0.1548	0.2429	0.3931	0.3119
**Stylo. PC1**	0.246	0.623		1.0000	<0.0001*	0.0008*	0.6963	0.0961	0.0508
**Stylo. PC2**	0.327	0.338	0.000		<0.0001*	0.0667	0.0687	0.9909	0.0319
**Gland PC1**	0.658	0.401	0.532	0.592		1.0000	0.0026*	0.1883	0.0002*
**Gland PC2**	0.070	-0.204	-0.457	0.261	0.000		0.2697	0.7766	0.9798
**SRO PC1**	0.498	-0.168	0.057	0.260	0.417	0.159		1.0000	<0.0001*
**SRO PC2**	0.019	-0.123	-0.238	-0.002	-0.189	-0.041	0.000		0.2275
**Flag. length**	0.335	0.146	0.278	0.304	0.498	-0.004	0.595	-0.174	

ML with or without phylogenetic uncertainty									
	**Dart PC1**	**Dart PC2**	**Stylo. PC1**	**Stylo. PC2**	**Gland PC1**	**Gland PC2**	**SRO PC1**	**SRO PC2**	**Flag. length**
**Dart PC1**		1.0000	0.3007	0.0111	<0.0001*	0.7575	0.0026*	0.1328	0.0133
**Dart PC2**	0.000		<0.0001*	0.0167	0.0056	0.1341	0.7651	0.3278	0.1318
**Stylo. PC1**	0.149	0.659		1.0000	0.0025*	0.0010*	0.7190	0.1012	0.1276
**Stylo. PC2**	0.356	0.337	0.000		<0.0001*	0.5025	0.0756	0.5732	0.0255
**Gland PC1**	0.638	0.386	0.419	0.651		1.0000	0.0027*	0.6739	0.0002*
**Gland PC2**	0.045	-0.215	-0.451	0.097	0.000		0.3104	0.9574	0.8661
**SRO PC1**	0.417	-0.043	0.052	0.254	0.416	0.146		1.0000	<0.0001*
**SRO PC2**	0.216	-0.141	-0.234	0.082	-0.061	0.008	0.000		0.7943
**Flag. length**	0.348	0.216	0.218	0.316	0.505	-0.024	0.558	-0.038	

Results are shown for the raw data and the analyses based on PICs calculated from the BI with phylogenetic uncertainty and the ML trees either with or without phylogenetic uncertainty. Note that the ML trees with or without uncertainty are identical. *, Significance after Bonferroni correction.
