# Supplementary material for: Shooting darts: co-evolution and counter-adaptation in hermaphroditic snails
Source: BMC Evol Biol. 2005 Mar 30;5:25. doi: 10.1186/1471-2148-5-25 (PMC1080126; doi:10.1186/1471-2148-5-25)
Supplement: Additional File 3 — Information about the location and collectors of the species used in the analysis. Numbers behind collector names represent the catalog number in the malacological collection of the Academy of Natural Sciences of Philadelphia. The EMBL accession numbers for the new and published DNA sequences are also included. Published sequences all derive from Wade et al. 2001 [25]. ‡, The specimens from this location were used for sequencing; § for these species the DNA sequence were available from EMBL but no specimens could be obtained for investigation. Therefore, the morphology data were based on Azuma 1995 [44]. [file 1471-2148-5-25-S3.pdf]

Additional file 3. Information about the location and collectors of the species used in the analysis.

Species	Location	Collector(s)	Accession number
Aegista vulgivaga (Schumacher & Boettger, 1890)	Honshu Island, Kyoto (Japan)	A. Gulick (A16693)	AY014139
Arianta arbustorum (Linnaeus, 1758)	Mittenwald (Germany)	N. Michiels & I. Vorndran	AY014136
	Luzern (Switzerland)	S. Schumo (A79833)	
Arion ater (Linnaeus, 1758)	Münster (Germany)	J. Koene	AY014144
Bradybaena similaris (Férussac, 1821)	Sendai (Japan)	S. Chiba	AY014138
Cantareus aperta (Born, 1778)	Roma (Italy)	C. Cavalieri	AY014129
Cantareus aspersus (Müller, 1774)	Amsterdam (The Netherlands)	F. Koene	AY014128
Cepaea hortensis (Müller, 1774)	Quebec City, QUE (Canada)	I. Picard	AY014131
Cepaea nemoralis (Linnaeus, 1758)	Münster (Germany)	J. Lange & J. Koene	AY014130
Cernuella cisalpina (Rossmässler, 1837)	Apulia (Italy)	D. Ferreri	AJ550955
Cernuella hydruntina (Kobelt, 1884)	Apulia (Italy)	D. Ferreri	AJ550957
Cernuella virgata (Da Costa, 1778)	Apulia (Italy)	D. Ferreri	AY014127
Chilostoma cingulatum (Studer, 1820)	Lucca (Italy)	M. Sosso	AJ550980
Chilostoma glaciale (Férussac, 1832)	Rocciamelone (Italy)	S. Schaparelli	AJ550979
Chilostoma planospira (Lamarck, 1822)	Lucca (Italy)	M. Sosso	AJ550981
Cochlicella acuta (Müller, 1774)	Roma (Italy)	C. Cavalieri	AY014126
Deroceras reticulatum (Müller, 1774)	Münster (Germany)	J. Koene	AY014119
Discus rotundatus (Müller, 1774)	Roma (Italy)	C. Cavalieri	AY014097
Eobania vermiculata (Müller, 1774)	Roma (Italy)	C. Cavalieri	AJ550977
	Lecce (Italy)	D. Ferreri	
	Nuoro, Sardegna (Italy) ‡	M. Sosso	
Euhadra amaliae (Kobelt, 1875)	(Japan)	M. Azuma §	AY014140
Euhadra quaesita (Dehayes, 1950)	Sendai (Japan)	A. Davison	AJ550970
Euhadra sandai (Kobelt, 1878)	(Japan)	M. Azuma §	AY014141
Fruticicola fruticum (Müller, 1774)	Münster (Germany)	J. Lange	AJ550971
Helicella itala (Linnaeus, 1758)	Bristol (United Kingdom)	J. Hutchinson	AJ550953
Helicigona lapicida (Linnaeus, 1758)	Bokova (Czech Republic)	H. Reise	AY014137
Helix lucorum taurica (Krynicki, 1833)	Crymea (Russia)	P. Balaban	AJ550975
Helix pomatia Linnaeus, 1758	Münster (Germany)	J. Koene	AJ550974
Helminthoglypta nickliniana (Lea, 1838)	San Mateo Co., CA (U.S.A.)	P. Liff-Grieff	AJ550973
Helminthoglypta tudiculata (Binney, 1843)	Wilmington, CA (U.S.A.)	P. Liff-Grieff	AJ550972
Humboldtiana nuevoleonis Pilsbry, 1927	Santiago, Nuevo Léon (México)	A. Correa	AJ550982
Hygromia cinctella (Draparnaud, 1801)	Bristol (United Kingdom)	J. Hutchinson	AJ550968
Leptaxis erubescens (Lowe, 1831)	Portela and Santa‡ (Madeira)	P. Van Riel	AJ550969

Species	Location	Collector(s)	Accession number
Marmorana scabriuscula (Deshayes, 1890)	Sicily (Italy)	F. Giusti	AY014133
Marmorana serpentina (Férussac, 1821)	Nuoro, Sardegna (Italy)	M. Sosso	AJ550978
Monacha cartusiana (Müller, 1774)	Roma (Italy) ‡	C. Cavalieri	AJ550962
	Washington D.C. (U.S.A.)	D. Prasher & D. Robinson	
Monacha parumcincta (Pfeiffer, 1837)	Lecce (Italy)	D. Ferreri	AJ550961
Monachoides vicinus (Rossmässler, 1842)	Dolni Lomná (Czech Republic)	H. Reise	AJ550967
Monadenia fidelis (Gray, 1834)	Humboldt Co., CA (U.S.A.)	D. Yoshimoto	AY014142
Otala lactea (Müller, 1774)	Montréal, QUE (Canada)	R. Chase	AJ550976
Perforatella bidentata (Gmelin, 1791)	Görlitz (Germany)	H. Reise & J. Hutchinson	AJ550966
Perforatella incarnata (Müller, 1774)	Görlitz (Germany)	H. Reise & J. Hutchinson	AJ550965
Polymita picta nigrolimbata Torre, 1950	Santiago de Cuba (Cuba)	B. Reyes Tur	AJ550960
Pseudotrichia rubiginosa (Rossmässler, 1838)	Münster (Germany)	J. Koene	AJ550963
Satsuma japonica (Pfeiffer, 1847)	(Japan)	M. Azuma §	AY014122
Theba pisana (Müller, 1774)	Roma (Italy)	C. Cavalieri	AY014135
	Lecce (Italy)	D. Ferreri	
	Nuoro, Sardegna (Italy)	M. Sosso	
Trichia hispida (Linnaeus, 1758)	Münster (Germany)	J. Koene	AY014125
Trichia striolata (Pfeiffer, 1828)	Quebec City, QUE (Canada)	I. Picard	AJ550964
	Bristol (United Kingdom) ‡	J. Hutchinson	
Vespericola columbiana (Lea, 1838)	Washington State (U.S.A.)	Unknown (A2425D)	AY014120
Xerarionta kellettii (Forbes, 1850)	Santa Catalina Island, CA (U.S.A.)	P. Liff-Grieff	AJ550959
Xeromunda durieui (Pfeiffer, 1848)	Lecce (Italy)	D. Ferreri	AJ550956
Xerosecta cespitum (Draparnaud, 1805)	Lucca (Italy)	M. Sosso	AJ550958
Xerotricha conspurcata (Draparnaud, 1801)	Apulia (Italy)	D. Ferreri	AJ550954

Numbers behind collector names represent the catalog number in the malacological collection of the Academy of Natural Sciences of Philadelphia. The EMBL accession numbers for the new and published DNA sequences are also included. Published sequences all derive from Wade et al. (2001). ‡, The specimens from this location were used for sequencing; §, for these species the DNA sequence were available from EMBL but no specimens could be obtained for investigation. Therefore, the morphology data were based on Azuma (1995).

References

Azuma M: *Colored Illustrations of the Land Snails of Japan.* Osaka: Hoikusha Publishing Co. Ltd.; 1995.

Wade CM, Mordan PB, Clarke B: **The phylogeny of the land snails (Gastropoda: Pulmonata).** *Proc. R. Soc. Lond. B* 2001, **268**:413- 422.
