# Supplementary material for: Shooting darts: co-evolution and counter-adaptation in hermaphroditic snails
Source: BMC Evol Biol. 2005 Mar 30;5:25. doi: 10.1186/1471-2148-5-25 (PMC1080126; doi:10.1186/1471-2148-5-25)
Supplement: Additional File 4 — Description of the data scored for the different reproductive organ characteristics. When traits are absent these are scored as zero. All relative sizes were measured on the camera lucida drawings. When the size is indicated relative to the reproductive organs this refers to the posterior reproductive organs (excluding the albumen gland) thus making sure that they are independent of the size of the penis and bursa tract. [file 1471-2148-5-25-S4.pdf]

Additional file 4. Description of the data scored for the different reproductive organ characteristics.

	**Love-dart**	
1.	Number	0, 1, 2, 3, 4, 5, 6, 7, 8
2.	Number of blades	0, 1, 2, 3, 4, 5, 6, 7, 8
3.	Perpendicular blades on blades	1=Present
4.	Length of blades (compared to total length)	0, 1/8, 1/4, 1/3, 1/2, 2/3, 3/4, 1
5.	Curvature	1=Slightly curved<br>2=Curved<br>3=Curved and twisted
	**Stylophore(s)**	
	**Functional stylophore(s)**	
1.	Number	0, 1, 2, 3, 4, 5, 6, 7, 8
2.	Size (relative to reproductive organs)	1=Small<br>2=Medium<br>3=Big
3.	Placement on vaginal duct	1=Near penis<br>2=Middle<br>3=Near bursa tract
	**Vestigial stylophore(s)**	
4.	Number	0, 1, 2, 3, 4, 5, 6, 7, 8
5.	Size (relative to functional stylophore)	1=Small (less than half)<br>2=Medium (half)<br>3=Big (same)
6.	Placement on vaginal duct	1=Above functional sac<br>2=Next to functional sac<br>3=Both
	**Glands**	
1.	Number	0, 1, 2, 3, 4, 5=many
2.	Size (relative to reproductive organs)	1=Small<br>2=Medium<br>3=Big<br>4=Very big (as long as spermoviduct)
3.	Type of branching	1=Tubular<br>2=Bifurcated<br>3=Branched<br>4=Branched and enlarged at end<br>5=Heavily branched<br>6=Flattened
4.	Placement	1=On (top of) stylophore<br>2=In base of stylophore<br>3=On vaginal duct, at base of stylophore<br>4=On vaginal duct, halfway to bursa tract<br>5=On vaginal duct, near bursa tract

Diverticulum of spermatophore-receiving organ		
1.	Presence	1=Yes
2.	Length (relative to bursa tract)	1=Shorter<br>2=Same<br>3=Less than twice as long<br>4=More than twice as long
3.	Placement (branching point from bursa tract)	1=Near vaginal duct<br>2=Middle of bursa tract<br>3=Near bursa copulatrix
4.	Length (relative to reproductive organs)	1=Shorter<br>2=Same<br>3=Less than twice as long<br>4=More than twice as long
**Flagellum**		
1.	Presence	1=Yes
2.	Length (relative to penis)	1=Shorter<br>2=Same<br>3=Less than twice as long<br>4=More than twice as long

When traits are absent these are scored as zero. All relative sizes were measured on the camera lucida drawings. When the size is indicated relative to the reproductive organs this refers to the posterior reproductive organs (excluding the albumen gland) thus making sure that they are independent of the size of the penis and bursa tract.
