# Supplementary material for: Shooting darts: co-evolution and counter-adaptation in hermaphroditic snails
Source: BMC Evol Biol. 2005 Mar 30;5:25. doi: 10.1186/1471-2148-5-25 (PMC1080126; doi:10.1186/1471-2148-5-25)
Supplement: Additional File 5 — The sequences for PCR and sequencing primers. Primers JOR58F, JOR59F, and JOR28R850 were used for PCR, whereby JOR59F was employed as the forward primer for Cernuella cisalpina and Euhadra quaesita, and JOR58F for all remaining species. Primers JOR28F50, JOR28F400, JOR28R401, JOR28F600, JOR28R601, and JOR28R850 were employed in sequencing reactions. [file 1471-2148-5-25-S5.pdf]

Additional file 5. The sequences for PCR and sequencing primers.

Primers	
**Forward** (situated in 5.8S rRNA gene)	
JOR58F	5' AACGCAAATGGCGGCCTCGG
JOR59F	5' CTCGGGTCCATCCCTCCGA
**Reverse** (located around position 850 of the 28S rRNA gene)	
JOR28R850	5' GAAGACGGGTCGGGTGGAATG
**Internal**	
JOR28F50	5' TCCGACCTCAGATCGGACGAG
JOR28F400	5' CTTTGAAGAGAGAGTTCAAGAG
JOR28R401	5' GCGGTTTCACGTACTCTTGAAC
JOR28F600	5' TACAAGCGGTTCGGHGGCGG
JOR28R601	5' AAGAAGGGCTGGTAGGGACGC

Primers JOR58F, JOR59F, and JOR28R850 were used for PCR, whereby JOR59F was employed as the forward primer for *Cernuella cisalpina* and *Euhadra quaesit*a, and JOR58F for all remaining species. Primers JOR28F50, JOR28F400, JOR28R401, JOR28F600, JOR28R601, and JOR28R850 were employed in sequencing reactions.
